# Berberine regulates mesangial cell proliferation and cell cycle to attenuate diabetic nephropathy through the PI3K/Akt/AS160/GLUT1 signalling pathway

**DOI:** 10.1111/jcmm.17167

**Published:** 2022-01-09

**Authors:** Wei‐Jian Ni, Xi‐Mei Guan, Jing Zeng, Hong Zhou, Xiao‐Ming Meng, Li‐Qin Tang

**Affiliations:** ^1^ Inflammation and Immune Mediated Diseases Laboratory of Anhui Province The Key Laboratory of Anti‐inflammatory of Immune Medicines (Ministry of Education) Anhui Institute of Innovative Drugs School of Pharmacy Anhui Medical University Hefei Anhui China; ^2^ Anhui Provincial Hospital The First Affiliated Hospital of USTC Division of Life Sciences and Medicine University of Science and Technology of China Hefei Anhui China; ^3^ Anhui Provincial Hospital Anhui Medical University Hefei Anhui China; ^4^ Department of Pharmacy Anhui Provincial Cancer Hospital The First Affiliated Hospital of USTC Division of Life Sciences and Medicine University of Science and Technology of China Hefei Anhui China

**Keywords:** berberine, cell cycle, diabetic nephropathy, glomerular mesangial cells, GLUT1, PI3K/Akt

## Abstract

High glucose (HG) is one of the basic factors of diabetic nephropathy (DN), which leads to high morbidity and disability. During DN, the expression of glomerular glucose transporter 1 (GLUT1) increases, but the relationship between HG and GLUT1 is unclear. Glomerular mesangial cells (GMCs) have multiple roles in HG‐induced DN. Here, we report prominent glomerular dysfunction, especially GMC abnormalities, in DN mice, which is closely related to GLUT1 alteration. In vivo studies have shown that BBR can alleviate pathological changes and abnormal renal function indicators of DN mice. In vitro, BBR (30, 60 and 90 μmol/L) not only increased the proportion of G1 phase cells but also reduced the proportion of S phase cells under HG conditions at different times. BBR (60 μmol/L) significantly reduced the expression of PI3K‐p85, p‐Akt, p‐AS160, membrane‐bound GLUT1 and cyclin D1, but had almost no effect on total protein. Furthermore, BBR significantly declined the glucose uptake and retarded cyclin D1‐mediated GMC cell cycle arrest in the G1 phase. This study demonstrated that BBR can inhibit the development of DN, which may be due to BBR inhibiting the PI3K/Akt/AS160/GLUT1 signalling pathway to regulate HG‐induced abnormal GMC proliferation and the cell cycle, supporting BBR as a potential therapeutic drug for DN.

## 1 INTRODUCTION

Diabetic nephropathy (DN) is a common and serious chronic kidney disease that is the main reason for the high mortality of diabetes and its expanding global burden.^1^ Current clinical treatment strategies can only delay its progression to a certain extent and cannot prevent its progression to end‐stage renal disease.^2^, ^3^ Meanwhile, authoritative research believes that abnormal cell status will cause a series of cascading effects, promote pathological changes and damage to the kidney, and accelerate the progression of renal dysfunction and DN.^4^ Among them, the abnormal proliferation of glomerular mesangial cells (GMCs) exists as early as the early stage of DN, and it plays an increasingly important role.^5^ However, the exact mechanism is still unclear, and there is no ideal strategy to prevent abnormalities in GMCs and the resulting disease development. Therefore, the specific mechanisms and effective treatment strategies need to be studied in depth.

High glucose (HG) is the basic factor that accelerates the occurrence and development of DN. In recent years, several glucose transporters have been found in glomeruli and cultured glomerular cells, such as facilitating glucose transporters (GLUT1, 3 and 4) and sodium‐glucose cotransporters (SGLTs).^6^, ^7^ The existing research mainly focuses on exploring the roles of SGLTs and promoting the clinical application of their inhibitors in the field of diabetes and its complications.^8^, ^9^ However, the role and mechanism of GLUT in the glomerulus are rarely mentioned. At the same time, multiple studies have found that hyperglycaemia can not only activate the abnormal proliferation of GMCs in DN animal models^10^ but also has similar effects in in vitro studies, which is worthy of attention.^11^ Moreover, abnormal expression of GLUT1 also appeared in the mesangial area stimulated by HG, but the specific mechanism has not been well elucidated.^12^ Based on the above research, we aimed to explore the changes and corresponding mechanisms of GLUT1 in GMCs stimulated by HG and in DN.

The phosphoinositide 3‐kinase (PI3K)/protein kinase B (Akt) pathway has been confirmed to be involved in many cellular processes, such as cell proliferation, differentiation, cell cycle progression and tumour growth.^13^ In the liver cancer, the PI3K/Akt pathway can activate GLUT1 signalling to regulate insulin‐dependent glucose metabolism.^14^, ^15^ During this process, some studies have found that AS160 (Akt substrate of 160 kDa/TbcId4), as a substrate of Akt, promotes GLUTs‐mediated glucose metabolism by binding to GLUTs (GLUT1 or GLUT4) vesicles and the plasma membrane, which provides power to activate the cell cycle alterations and cell proliferation.^16^, ^17^ Our previous research found that HG can activate the PI3K/Akt signalling of podocytes to promote the development of DN.^18^ In addition, preliminary research results indicate that HG conditions can cause abnormal changes in GMCs, and at this time the expression of GLUT1 is also significantly increased. In view of this, further in‐depth research is needed to investigate the relationship among HG‐induced abnormal GMC proliferation, the PI3K/Akt pathway and GLUT1 alterations in DN.

In recent years, traditional Chinese medicine has attracted increasing attention due to its outstanding performance in treating SARS‐CoV‐2‐induced pneumonia, cardio‐/cerebrovascular diseases and diabetes and its complications.^19^, ^20^, ^21^ Among these, berberine (BBR), a famous herb extract generally extracted from *rhizoma coptidis* rhizomes and cortex *phellodendri*, has been reported to have potent renoprotective effects.^22^ Multiple studies have shown that BBR can not only reduce hyperglycaemia, regulate dyslipidaemia and attenuate kidney inflammation but also improve insulin resistance and enhance insulin activity in an animal model of diabetes.^22^, ^23^ We previously reported that BBR can enhance autophagy by inhibiting PI3K/Akt signalling, thereby reducing glomerular podocyte injury in streptozocin (STZ)‐induced DN.^18^ However, the effect and underlying mechanism of BBR on GMCs in DN remain to be further studied. Therefore, the present study aimed to evaluate the effect of BBR on abnormal GMC proliferation in DN and to further explore the underlying mechanism and relationship among BBR, abnormal GMC proliferation, the PI3K/Akt pathway and GLUT1 alterations, which will offer insights into controlling cellular responses to hyperglycaemia that initiate the progression of DN.

## 2 MATERIALS AND METHODS

### 2.1 Materials

Streptozocin (S0130) and D‐glucose (G8270) were obtained from Sigma‐Aldrich Chemical Company (Sigma). BBR (BWC9020‐2016) was procured from BeNa Biotechnology. Membrane and cytosolic protein extraction kits (P0033), trypan blue (ST798), EdU (5‐ethynyl‐2′‐deoxyuridine) assays (C0071S) and cell cycle detection kits (BB‐4104) were acquired from Beyotime Biotechnology. The 2‐NBDG (2‐deoxy‐2‐ [(7‐nitro‐2,1,3‐benzoxadiazol‐4‐yl) amino]‐D‐glucose) assay (N13195) was obtained from Thermo Fisher Scientific. Anti‐PI3K‐p85 (#4292), anti‐Akt (4691S), anti‐p‐Akt (#4060), anti‐AS160 (#2670), anti‐p‐AS160 (#8619) and anti‐GAPDH (#5174) antibodies were obtained from Cell Signaling Biotechnology. Rabbit anti‐GLUT1 (ab115730) and anti‐Cyclin D1 (ab16663) antibodies were purchased from Abcam Biotechnology (Abcam).

### 2.2 Animals model and experimental design

Male C57BL/6 mice (6–8 weeks) were purchased from the Experimental Animal Centre of Anhui Medical University (Hefei, China) and provided with adaptable feeding (free access to standard food and water, 22 ± 2°C and humidity of 60 ± 5%) for 7 days. The mice were randomly divided into five groups: normal control (Control or NC) group; STZ + high glucose/fat diet (DN) group; DN + BBR (90, 180 mg/kg) ((BBR (90 mg/kg), BBR (180 mg/kg)) groups and DN + metformin (200 mg/kg) (Metformin (200 mg/kg)) group. After adaptive feeding, the type 2 DN model was established in accordance with the accepted method (https://www.diacomp.org/shared/protocols.aspx). Mice in the drug treatment groups were administered BBR (90 and 180 mg/kg) and metformin (200 mg/kg) by intragastric gavage every other day for 12 weeks. The control and DN groups were given the same amount of saline intragastrically. At the end of 12 weeks, 24 h urine samples were collected from all mice using the metabolic cage. Blood was collected from the mice under anaesthesia in a fasted state. Then, kidney samples were harvested and rapidly frozen in liquid nitrogen or fixed in 4% paraformaldehyde solution and 2.5% glutaraldehyde solution for subsequent experiments.

### 2.3 Biochemical analyses

Collected blood was centrifuged for testing of blood urea nitrogen (BUN), serum creatinine (Scr) and the ratio of urine total protein (UTP) using an automated biochemistry analyser (Model 7600 Series Automatic Analyzer, Hitachi Corporation) as described previously.^24^

### 2.4 Kidney histopathology

The fixed kidney tissues were embedded in paraffin and cross‐sectioned (4 μm) for histological examination. Haematoxylin and eosin and periodic acid‐Schiff (PAS) staining were performed as described previously.^25^ Finally, the mesangial expansion index and tubular‐interstitial injury index of renal tissue were evaluated from six randomly selected fields.^26^

### 2.5 Transmission electron microscopy

Kidney tissue was fixed in 2.5% glutaraldehyde and 1% osmic acid at 4°C. Sections were with 1% uranyl acetate and embedded in epoxy resin (EPON) polymerized in gelatin capsules at 60°C for 48 h.^26^ The sections were observed using a transmission electron microscope (JEM1400, Hitachi).

### 2.6 Immunohistochemistry and immunohistofluorescence

Renal sections were heated in a microwave at 60°C for 2 h and incubated with 3% hydrogen peroxide. The paraffin tissue sections were treated with anti‐GLUT1 (1:250) and anti‐Cyclin D1 (1:200) antibodies for 24 h at 4°C, followed by secondary antibodies (goat anti‐rabbit IgG/H&L for 30 min and rabbit immunoglobulin G/Cy3 for 60 min) at 37°C. After staining with DAB for 5 min and DAPI for 5–8 min, the slides were observed under a microscope (Zeiss Spot, Carl Zeiss).

### 2.7 Cell culture, MTT (thiazole blue colorimetry) assay and trypan blue

Cultivation of GMCs according to established methods. Serial passaging was performed when the cells reached 75% confluence. The confluent GMCs were grown in serum‐free DMEM for 12 h prior to the following experiments.^25^ GMCs (1.0 × 10^4^ cells/well) were seeded into 96‐well plates and incubated at 37°C overnight. After that, cells were pretreated with serum‐free medium for 12 h and subsequently cultured with serum‐free normal glucose (D‐glucose, 5.5 mmol/L) medium, serum‐free HG (D‐glucose, 30 mmol/L) or serum‐free HG plus different concentrations of BBR (7.5, 15, 30, 60, 90, 120 and 150 μmol/L) for different durations (12, 24, 36 and 48 h, respectively). Finally, MTT solution (5 mg/ml) was added to plates, and the samples were incubated for 4–6 h. The optical density (OD) of each well was measured using a microplate reader (SpectraMax i3x, Molecular Devices) at 490 nm according to the operations manual. Cell viability was expressed as the percentage of viable cells relative to that of nontreated control cells. Meantime, GMCs were cultured under HG (30 mmol/L) concentration condition and treated with BBR at different times. After BBR treatment, trypan blue was used to detect the cell survival.^25^

### 2.8 Flow cytometry

Glomerular mesangial cells were washed twice with cold phosphate buffer solution (PBS) and fixed in cold 70% ethanol overnight at 4°C. Fixed cells were washed with cooled PBS and stained using a cell cycle assay kit. The experiment was repeated three times. Finally, samples were analysed on a Beckman Coulter instrument, and data were collected for 10,000 single‐cell events. The percentage of cells in the G1, S and G2 phases of the cell cycle was determined by CytExpert software. Finally, data are presented as histograms.

### 2.9 Fluorescent EdU

Glomerular mesangial cells at a density of 1 × 10^4^ cells/well were cultured in 24‐well plates for 24 h, and then the EdU assay was performed using fluorescence microscopy (DM2000, Leica) in different groups. Briefly, the cells were fixed with 4% paraformaldehyde (PFA) for 15 min at room temperature, and then permeabilized with 0.3% Triton X‐100 for 15 min. Then, the click reaction solution was added and samples were incubated for 30 min in the dark. After staining with Hoechst solution for 10 min, images were obtained with a microscope and were analysed with Image‐Pro Plus. The EdU incorporation rate was calculated as the ratio of EdU‐positive cells (green cells) to total Hoechst‐positive cells (blue cells).

### 2.10 Western blot

To examine the translocation of GLUT1, we used Membrane and Cytosol Protein Extraction Kit to extract and isolate its cytoplasmic and membrane proteins. Cell and tissue total proteins were extracted or isolated according to the manufacturer protocols. Meanwhile, detect the expression of various proteins according to the previously established western blot experimental conditions^27^ with the appropriate primary antibodies: anti‐PI3K‐p85 (1:1000), anti‐Akt (1:1000), anti‐p‐Akt (1:1000), anti‐AS160 (1:1000), anti‐p‐AS160 (1:1000), anti‐GLUT1 (1:1000) and secondary antibody. All of the immunoblots were detected with HRP western blotting detection reagents (Millipore Corporation). Equal loading was confirmed using anti‐GAPDH or anti‐β‐actin antibody (1:1000).

### 2.11 RT‐qPCR analysis

To detect the relative mRNA level, a Transcription First Strand cDNA Synthesis kit (Vazyme Biotech) was used to perform the reverse transcription. The reaction conditions were as follows: 50℃ for 15 min and 85℃ for 5 s. A PCR reaction system was prepared using SYBR^®^‐Green Real‐Time PCR Master mix (Thermo Fisher Science, ABI7500). The PCR reaction conditions were as follows: 95℃ for 10 s, followed by 40 cycles of 10 s at 95℃ and 30 s at 60℃. GAPDH served as an internal control to normalize the relative expression of GAPDH, PI3K, Akt, GLUT1 and AS160. All data were quantified using the $2^{‐\Delta \Delta C_{t}}$ method and run‐in triplicate for each sample. The primer sequences used in RT‐qPCR are listed in Table 1.

**TABLE 1:** The RT‐qPCR primer sequences (Mouse)

Gene name	Forward primer	Reverse primer
GAPDH	AGGTCGGTGTGAACGGATTTG	GGGGTCGTTGATGGCAACA
PI3K‐p85	ACACCACGGTTTGGACTATGG	GGCTACAGTAGTGGGCTTGG
Akt	ATGAACGACGTAGCCATTGTG	TTGTAGCCAATAAAGGTGCCAT
GLUT1	CAGTTCGGCTATAACACTGGTG	GCCCCCGACAGAGAAGATG
AS160	GAGTCGCCTAGCTGCATTCAG	CCACGTACCATAGCCGGAA

### 2.12 2‐NBDG assay for glucose uptake

Briefly, GMCs were seeded at a concentration of 1 × 10^5^ cells/ml/well in duplicate in a six well plate and incubated overnight at 37℃. The cells were washed twice with cold KBR after HG treatment and then cultured with 2‐NBDG for 30 min at 37°C. Finally, the cells were washed twice with cold KBR buffer to halt the glucose uptake and then resuspended in KBR for fluorescence detection using a flow cytometer at a fluorescence excitation wavelength of 488 nm and emission wavelength of 520 nm.

### 2.13 Statistical analysis

Data were assessed using SPSS 23.0 (IBM Corporation). Significant differences were evaluated using one‐way ANOVA with a post hoc Bonferroni correction (GraphPad Prism 5.0; GraphPad Software). A two‐sided *p*‐value <0.05 was considered significant. The data are presented as the mean ± SEM.

## 3 RESULTS

### 3.1 BBR ameliorates renal pathological changes in a DN mouse model

**FIGURE 1:**
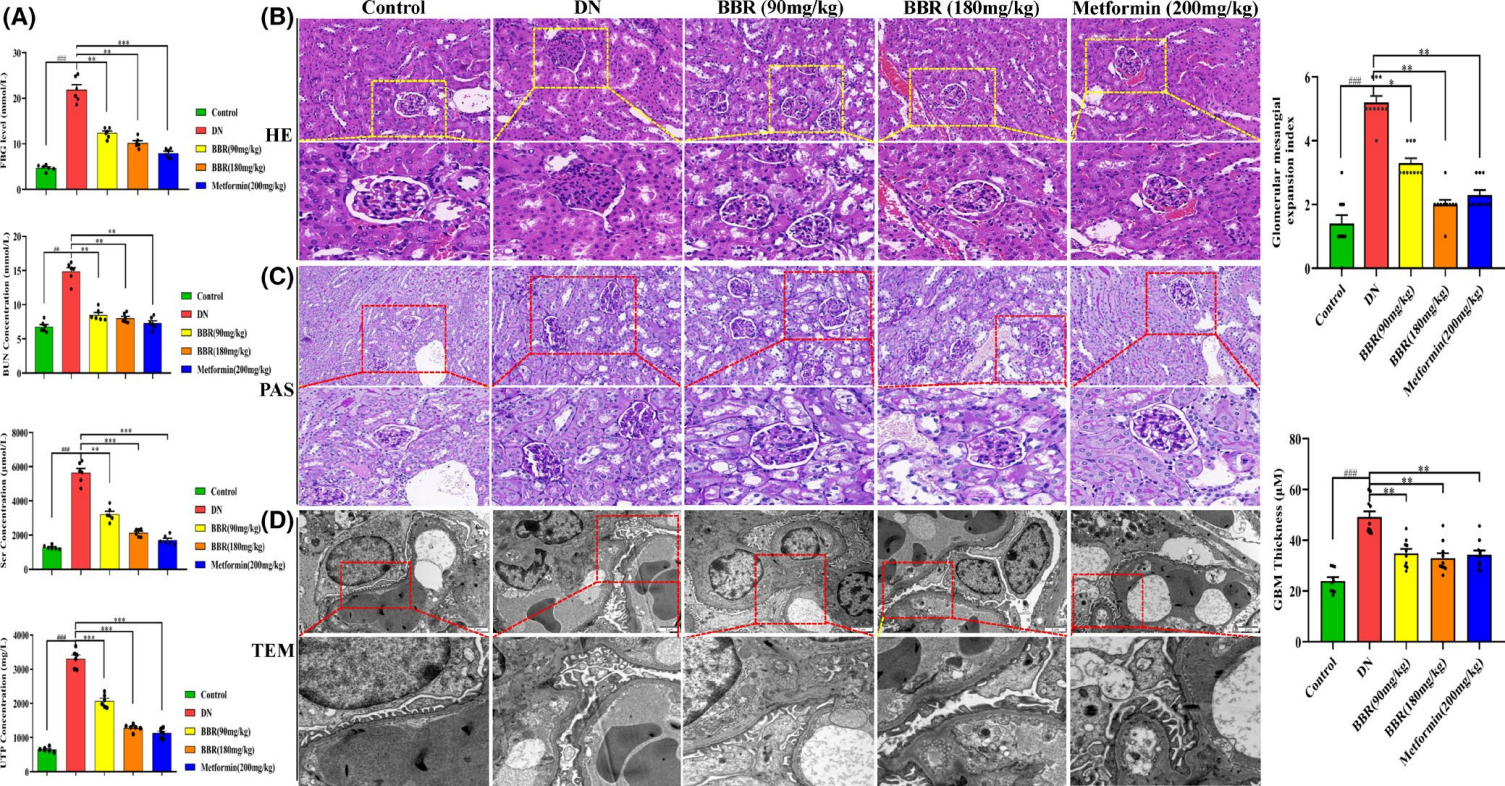
Effect of BBR on the renal morphological and functional changes in DN mice. The STZ‐induced diabetic mice were intragastrically administered with BBR (90 and 180 mg/kg) and metformin (200 mg/kg) once daily for 12 weeks. The pathological analysis of glomerular was performed by haematoxylin and eosin staining and periodic acid‐Schiff staining (PAS). The glomerular ultrastructure of DN mice was observed by transmission electron microscopy (TEM). Meantime, levels of fasting blood glucose (FBG), serum creatinine (Scr), blood urea nitrogen (BUN) and total urine protein (UTP) levels were measured. (A) Renal functional biochemical indexes changes; (B) haematoxylin and eosin staining; (C) PAS staining and (D) TEM of the basement membrane thickening. Images were taken at 40× magnification, and metformin was considered as positive medicine. The data are expressed as Mean ± SEM, *n* = 6, ^##^ *p* < 0.01 and ^###^ *p* < 0.001 vs. Control group, *p < 0.05 and **p < 0.01 and ***p < 0.001 vs. DN group

To test whether berberine provides renal protection in the high‐fat/sugar diet with STZ‐induced DN model, diabetic mice were given berberine (90 and 180 mg/kg) by gavage, and metformin (200 mg/kg) was used as the positive medicine. A variety of blood and urine biochemical indicators, including FBG, BUN, Scr and UTP, in DN mice were significantly increased. Moreover, these changes were remarkably reduced by treatment with the two dosages of BBR and metformin (Figure 1A). As shown in Figure 1B, Haematoxylin and eosin staining showed unclear glomerular structure, mesangial cell proliferation, glomerular basement membrane (GBM) thickening and a certain degree of fibrosis accumulation in DN mice compared with control mice. When treated with BBR (90 and 180 mg/kg), the pathological changes, such as GBM thickening and the unclear glomerular structure, were distinct and mesangial expansion was also extremely reduced. However, the glomerular sclerosis can still be observed to some extent. Meanwhile, metformin (200 mg/kg) treatment partially improved the aforementioned histological features. PAS staining was used to examine the accumulation of mesangial matrix and mesangial expansion in mouse tissue, as its components were mainly composed of polysaccharide‐associated ingredients. The DN group exhibited a larger PAS‐positive area and mesangial expansion than the control group at the 12th week (Figure 1C). The BBR (180 mg/kg) and metformin (200 mg/kg) groups showed decreased PAS‐positive areas and mesangial expansion compared with the DN group. Meanwhile, the PAS‐positive areas and mesangial expansion of the mice in the BBR (90 mg/kg) treatment group were reduced to a lesser extent than those in the other drug treatment groups. TEM observations showed no obvious hyperplasia in the basement membrane thickness in the mouse tissue of the control group. Podocytes were not seen as obvious abnormalities, and the morphological structure was well balanced. Compared with the control group, the GBM was obviously thickened, podocyte foot process fusion was observed and the arrangement was disordered in the DN group. BBR (90 and 180 mg/kg) and metformin (200 mg/kg) treatment normalized the aforementioned abnormal alterations in the DN group to a certain degree (Figure 1D).

### 3.2 BBR changes the PI3K/Akt pathway in DN mice

**FIGURE 2:**
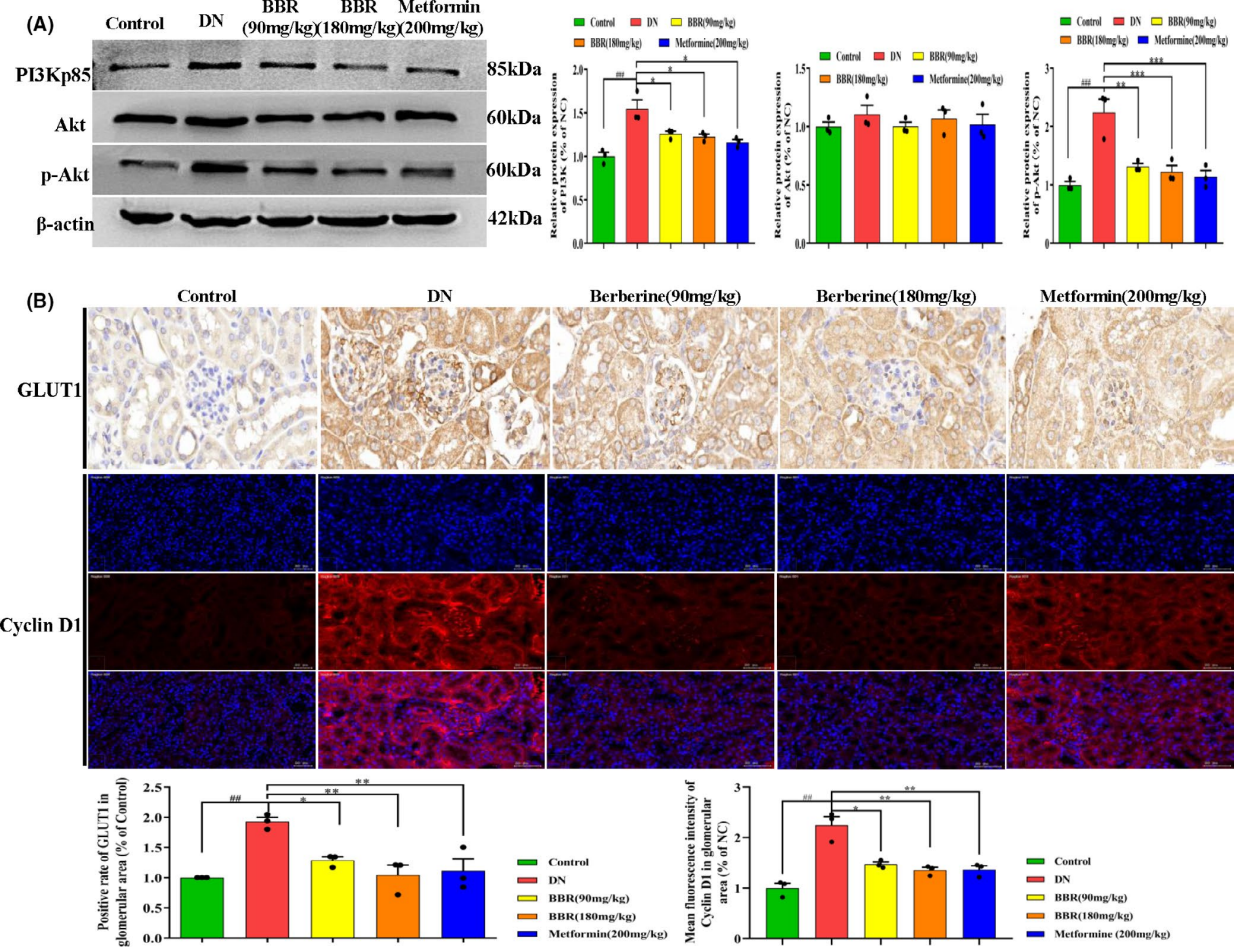
Effect of BBR on PI3K/Akt pathway and GLUT1 expression in kidney tissue of DN mice. Mice were subjected to DN, and orally administered with BBR (90 and 180 mg/kg) or metformin (200 mg/kg). (A) Representative western blot and the right lane showing the densitometry measurements normalized to β‐actin. The data represent the mean ± SEM, *n* = 3. (B) Relative expression of GLUT1 and cyclin D1 in kidney tissues by immunohistochemistry and immunohistofluorescence. The data are displayed in the form of Mean ± SEM with *n* = 3/6, ^##^ *p* < 0.01, ^###^ *p* < 0.001 vs. Control group, *p < 0.05, **p < 0.01, ***p < 0.001 vs. DN group

As shown in Figure 2, the expression of PI3K‐p85 and p‐Akt was significantly increased in DN mice compared with control mice, while changes in Akt were undistinguishable. Statistical analysis indicated that treating with different dosages of BBR can markedly downregulate the expression of PI3K‐p85 and p‐Akt. Western blot analysis demonstrated that the PI3K/Akt pathway was activated in DN mice. Notably, BBR inhibited the activity of the PI3K/Akt pathway (Figure 2A).

### 3.3 BBR inhibits the expression of GLUT1 and Cyclin D1 in the kidney tissue of DN mice

The immunohistochemistry and immunohistofluorescence results indicated that GLUT1 and Cyclin D1 were expressed in glomerular and tubule areas (Figure 2B). Semi‐quantitative analysis revealed an obvious expression of GLUT1 and Cyclin D1 in the DN group compared with the control group (*p* < 0.01). The change in Cyclin D1 was also confirmed by Western blot. Treatment with BBR (90 and 180 mg/kg) alleviated this phenomenon, and the same effect occurred in the metformin (200 mg/kg) treatment group.

### 3.4 BBR reverses HG‐induced abnormal GMCs proliferation

**FIGURE 3:**
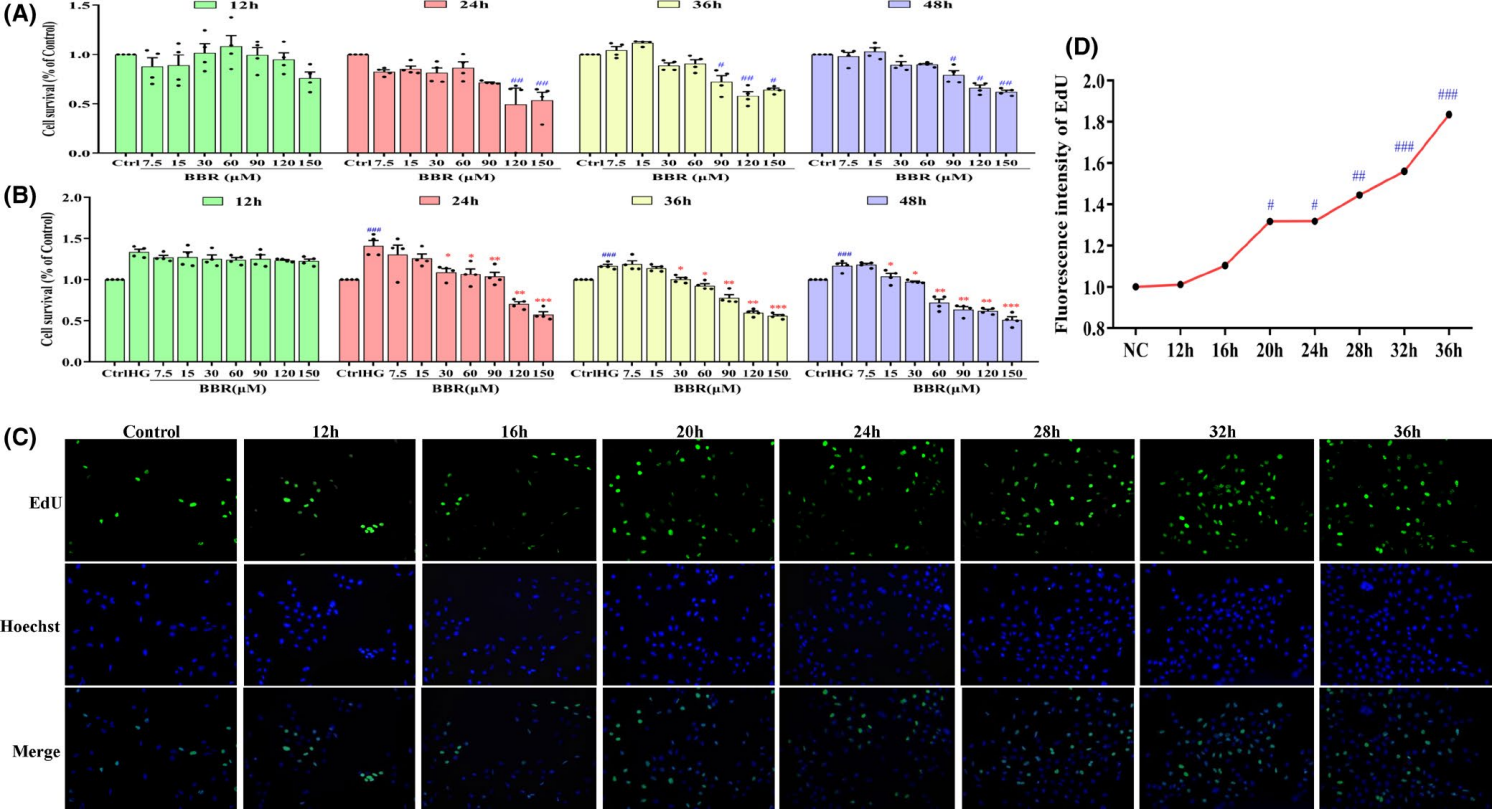
Effect of BBR and HG on GMCs proliferation and cell cycle redistribution at different times. (A) Effect of different concentrations of BBR on the activity of normal GMCs at different times; (B) Effect of different concentrations of BBR on GMCs proliferation in HG at different times. The data represent the mean ± SEM, *n* = 6; (C) Representative EdU staining image of GMCs treated with high glucose was shown at 0, 12, 16, 20, 24, 28, 32 and 36 h; (D) The quantification results of EdU‐positive cells (%). The data are expressed as Mean ± SEM, *n* = 6/3, ^#^ *p* < 0.05, ^##^ *p* < 0.01, ^###^ *p* < 0.001 vs. Control group; *p < 0.05, **p < 0.01, ***p < 0.001 vs. HG. HG, high glucose

Glomerular mesangial cells were used to establish models under HG conditions (30 mmol/L), while those under normal glucose conditions (5.5 mmol/L) were used as controls. Then, MTT assay and trypan blue staining were used to determine the cell proliferation characteristics and screen the appropriate BBR intervention concentration and time. From the results of MTT and trypan blue staining, HG significantly promoted the abnormal GMC proliferation at 24 h, and this situation continued with time (24 h→48 h) (*p* < 0.001) (Figure 3B), while the above phenomenon was not observed in the control group (Figure 3A). After considering the inhibitory effect, BBR (30, 60 and 90 μmol/L) were considered the appropriate candidate concentrations for the first 24 h of treatment (Figure 3B) and cytotoxicity effect is shown in Table S1. To precisely understand the abnormal GMCs proliferation characteristics and choose the intervention time for the next step, we used EdU assay to detect and find that the unapparent and apparent abnormal GMCs proliferation occurs at 20 h (*p* < 0.05), and therefore, 12, 16, 20, 24 and 28 h intervention times are included in the following experiment (Figure 3C,D).

### 3.5 BBR regulates cell cycle redistribution of GMCs

**FIGURE 4:**
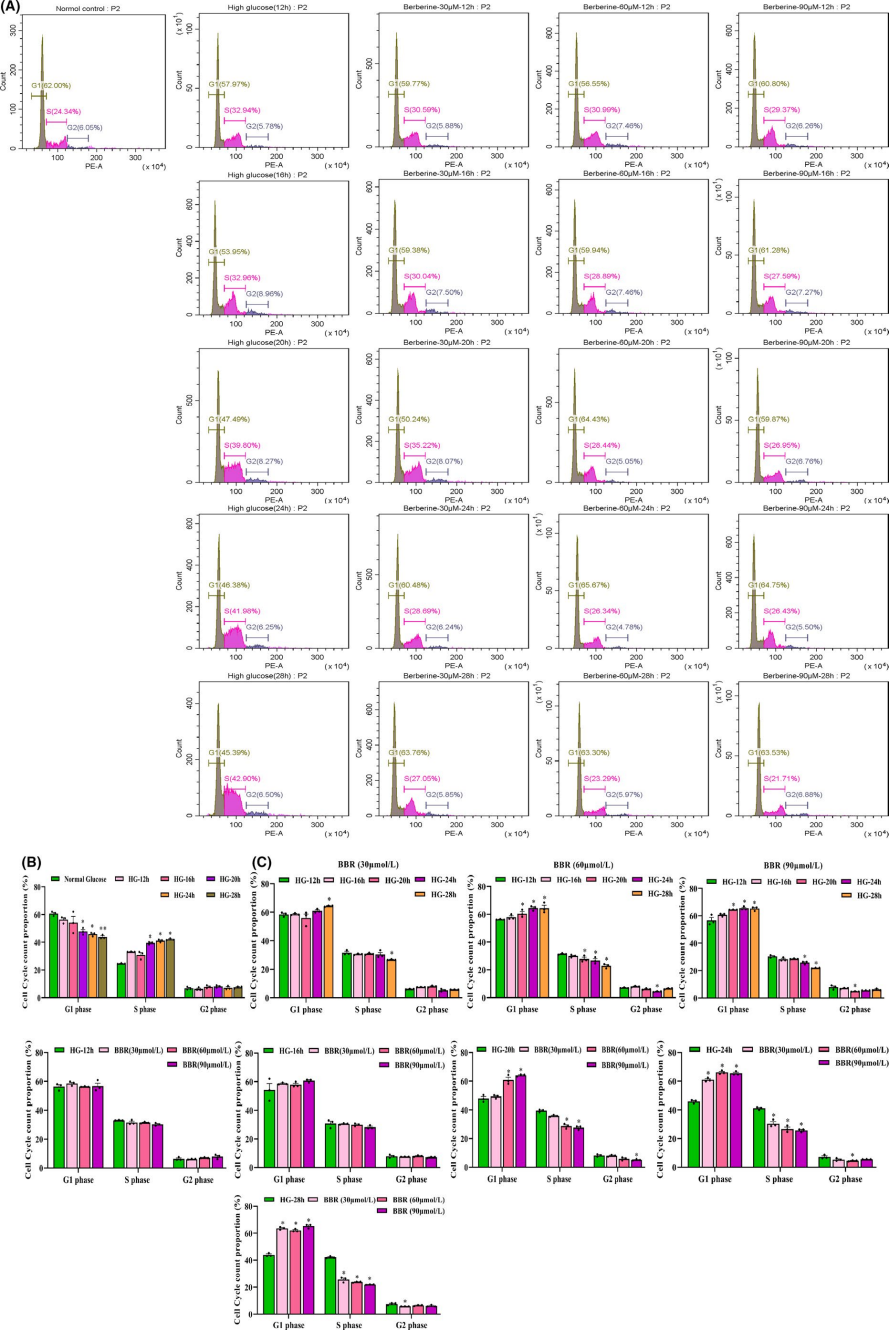
HG‐induced GMCs cell cycle distribution at different times. (A) The cell cycle of HG‐induced GMCs after different concentration of BBR treatment at different times; (B) Effect of high glucose in GMCs cell cycle; (C,D) Effect of BBR on HG‐induced GMCs cell cycle redistribution at different times. The results are expressed as mean ± SEM, *p < 0.05, **p < 0.01, vs. Normal glucose in Figure 4B; *p < 0.05, vs. HG–12h in Figure 4C,4D. HG, high glucose

In this section, we used flow cytometry to explore the effect of HG and BBR on regulating the cell cycle redistribution of GMCs (Figure 4A). The results showed that HG significantly prevented GMCs from entering G1 phase (*p* < 0.05) and accelerated cells from entering S phase (*p* < 0.001), while HG had no remarkable influence on G2 phase cells (*p* > 0.05) (Figure 4B). After treatment at 12 and 16 h, almost no cell cycle redistribution changes were detected in GMCs (*p* > 0.05). As shown in Figure 4C, BBR (60 and 90 μmol/L) not only increased the proportion of GMCs in G1 phase but also reduced the proportion of cells in S phase at 20 h, and this tendency became more significant over time (*p* < 0.001). After 28 h, the same phenomenon occurred in the BBR (30 μmol/L) group (*p* < 0.001), as shown in Figure 4D. Therefore, BBR (60 μmol/L) was selected as the most appropriate intervention concentration in the next steps after comprehensive consideration.

### 3.6 BBR regulates PI3K/Akt/AS160/GLUT1 pathway

**FIGURE 5:**
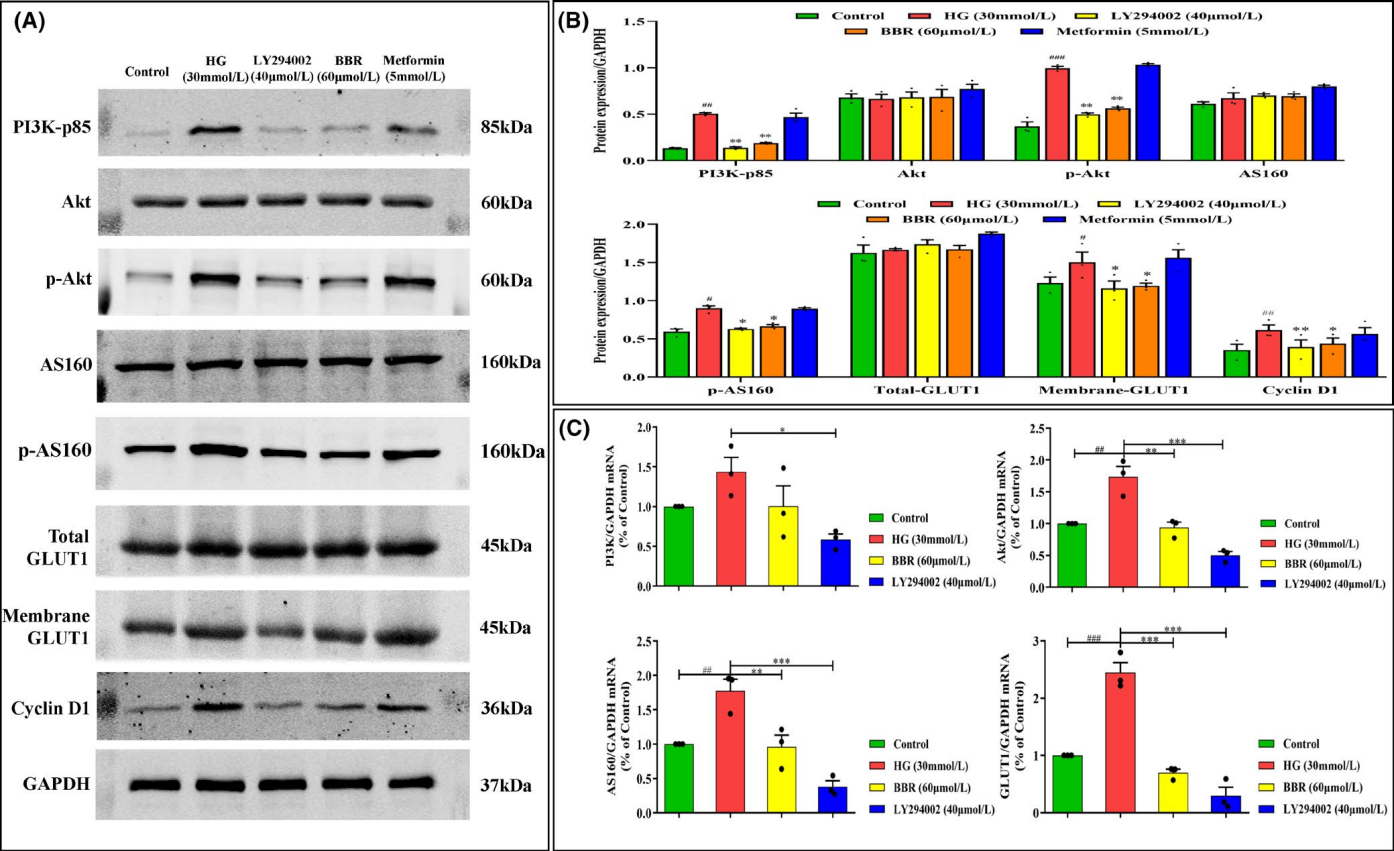
Effect of BBR on regulating the PI3K/Akt/AS160/GLUT1 pathway. Protein was extracted from cultured GMCs. GAPDH was used as internal controls. Intensity of PI3K, AKT, p‐AKT, AS160, p‐AS160, Total GLUT1 and m‐GLUT1 was quantified, normalized to internal control and expressed as mean ± SEM. (A,B) The levels of PI3K, Akt, p‐Akt, AS160, p‐AS160, Total GLUT1 and m‐GLUT1 were detected in each group; (C) RT‐qPCR analyses were performed to detect the mRNA expression of PI3K, Akt, AS160 and GLUT1 in each group. Relative expression of PI3K, Akt, AS160 and GLUT1. Data are expressed as Mean ± SEM, *n* = 3. ^#^ *p* < 0.05, ^##^ *p* < 0.01, ^###^ *p* < 0.001 vs. Control group; *p < 0.05, **p < 0.01, ***p < 0.001 vs. HG group. HG: high glucose; BBR: HG + BBR (60 μmol/L); LY294002 (HG + LY294002); Metformin: HG + metformin (5 mmol/L)

When exploring the effect of BBR on the PI3K/Akt/AS160/GLUT1 pathway, BBR (60 μmol/L) and LY294002 (40 μmol/L) were selected as appropriate intervention concentrations and an appropriate PI3K inhibitor respectively. As shown in Figure 5A,B, BBR (60 μmol/L) treatment significantly inhibited the HG‐induced increase in the protein levels of PI3K‐p85, phosphorylated Akt (p‐Akt), phosphorylated AS160 (p‐AS160) and membrane GLUT1 but had little effect on their total protein expression at 20 h. Moreover, BBR also suppressed the mRNA expression of these parameters, as shown by the qPCR results (Figure 5C). These results indicated that BBR has a role similar to that of the PI3K inhibitor (LY294002, 40 μmol/L) that inhibits the signal transduction of the PI3K/Akt/AS160/GLUT1 pathway in GMCs under HG conditions.

### 3.7 BBR affects GLUT1‐mediated glucose uptake, cell cycle redistribution and abnormal GMCs proliferation

**FIGURE 6:**
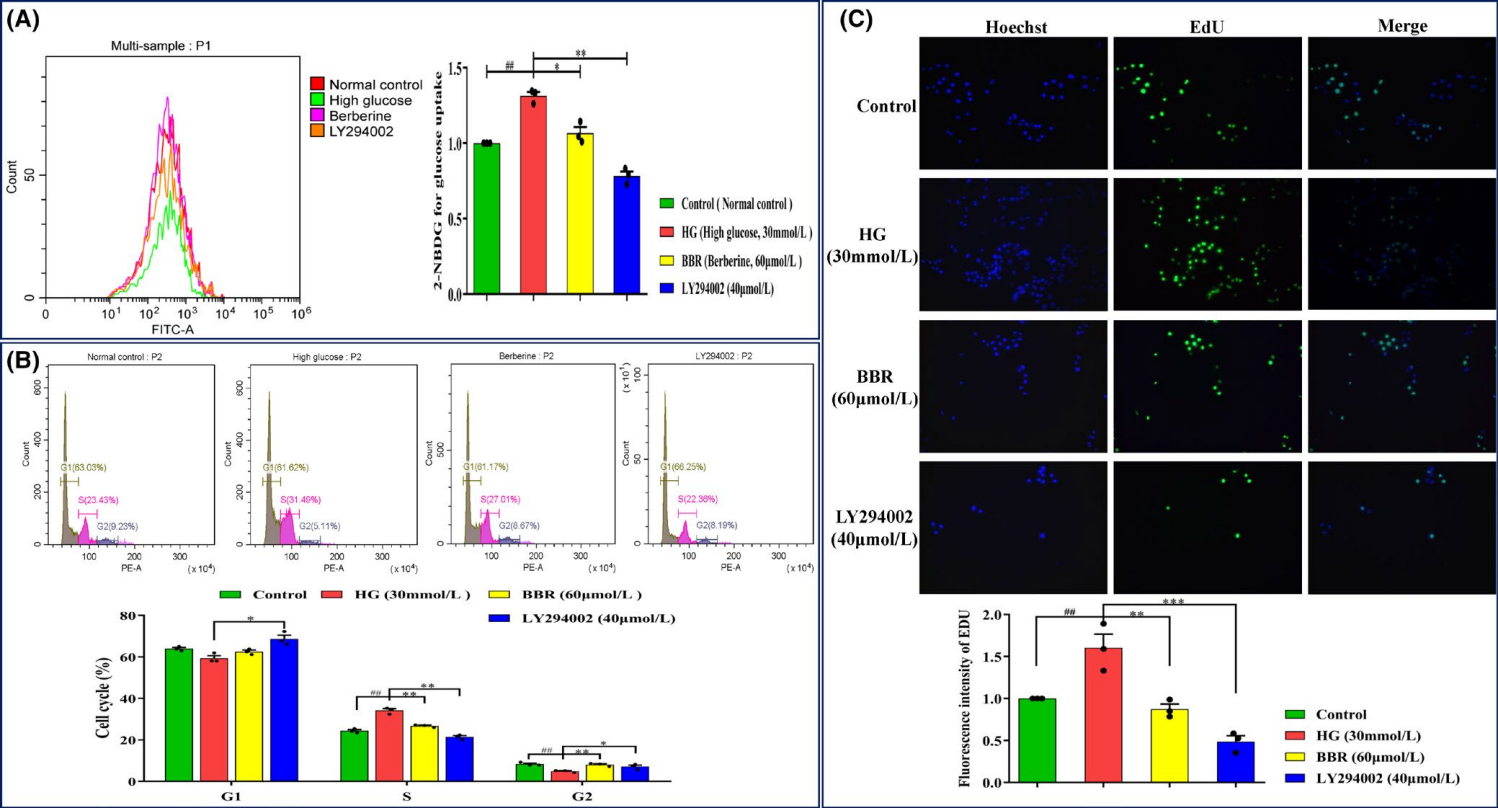
HG‐induced GMCs cell cycle redistribution at different times. (A) The representative images of 2‐NBDG detected by flow cytometry and the results of 20 h GMC glucose uptake under HG conditions. (B) PI staining to detect GMC cell cycle proportion (G1 phase, S phase and G2 phase) during HG stimulation and BBR treatment. (C) Quantitative analysis results of EdU staining‐positive cells. Data are expressed as Mean ± SEM, *n* = 3. ^##^ *p* < 0.01, ^###^ *p* < 0.001 vs. Control group; *p < 0.05, **p < 0.01, ***p < 0.001 vs. HG group. HG: high glucose; BBR: HG + BBR (60 μmol/L); LY294002 (HG+LY294002)

Based on the above results, BBR can inhibit the signal transduction of the PI3K/Akt/AS160/GLUT1 pathway to reduce the level of membrane GLUT1. In Figure 6A, a 2‐NBDG assay was used to measure the glucose uptake, and the results indicated that HG significantly increased the glucose uptake of GMCs (*p* < 0.01), while this effect was reversed by BBR (60 μmol/L) and LY294002 (40 μmol/L) treatment (*p* < 0.05 and *p* < 0.01, respectively). Moreover, BBR (60 μmol/L) markedly regulated the cell cycle redistribution and inhibited abnormal cell proliferation at 20 h. Specifically, BBR not only increased the proportion of G1 phase cells but also arrested the GMCs in G1 phase from entering S phase (Figure 6B) (*p* < 0.01), which notably inhibited abnormal GMCs proliferation under HG conditions (*p* < 0.01) (Figure 6C).

## 4 DISCUSSION

The present study explored the renoprotective role of BBR in mouse GMCs. Changes in biochemical indicators in DN mice showed a significant reduction in FBG, BUN, Scr and UTP after treatment with BBR. In addition, the DN mice exhibited exacerbation of kidney pathology changes, such as mesangial matrix accumulation, mesangial area expansion and glomerular hypertrophy, which indicated abnormal GMC proliferation, and BBR showed an ameliorating effect in these mice at 12 weeks. Moreover, excessive glycogen deposition demonstrated excessive glucose metabolism in the glomerular area compared with that of control mice, and BBR reduced glycogen deposition. Transmission electron microscopy results also show that berberine can significantly reduce the GBM thickening and foot process fusion. Given all this, hyperglycaemia may induce additional glucose uptake, which contributes to dysfunction of GMCs, eventually causing glomerular damage and abnormal kidney function.^28^ When treated with BBR, these abnormal changes can be significantly improved, and the effect is similar to metformin, which indicates that BBR is likely to exert its renoprotective effect through its glucose regulation function.

Some studies have demonstrated that the GLUT1‐modulated glucose metabolism can be activated and regulated by the PI3K/Akt pathway.^29^, ^30^, ^31^ During this process, the Akt 160 kDa/TbcId4 substrate, named AS160, can bind to GLUT1 vesicles and the plasma membrane to regulate glucose metabolism and provide power to activate cell cycle alterations and cell proliferation.^32^ In the current study, the high expression of PI3K and p‐Akt in DN mice indicated the activation of the PI3K/Akt signalling pathway. PI3K/Akt signalling pathway activation causes increased expression of GLUT1 and cyclin D1 in the glomerular area, indicating that PI3K/Akt can promote abnormal cell cycle changes and cell proliferation in the glomerulus by increasing GLUT1‐mediated glucose transport. To explore the mechanism of BBR on the above changes, multiple in vitro studies have been implemented, and the results showed that HG can significantly promote abnormal GMC proliferation at 24 h compared to 12 h and normal glucose, which indicated that HG is an important factor for abnormal GMC proliferation and that the induction phenomenon requires an initiating process. Additionally, BBR exerted an ideal inhibitory effect on HG‐induced GMC proliferation in the concentration range from 30 to 90 μmol/L after adjusting for its cytotoxicity. The aforementioned data revealed that the appropriate concentration of BBR (30, 60 and 90 μmol/L) could effectively inhibit HG‐induced abnormal GMC proliferation. Cellular cycle stability is a critical factor that affects cell functions, particularly growth, proliferation and differentiation, and is not limited to the embryological state.^33^ As the research continued, we found that the GMC cell cycle changed undifferentially before 20 h of HG stimulation. When GMCs were exposed to HG for more than 20 h, HG significantly increased the percentage of cells entering the S phase while reducing the number of cells in G1 phase but had no effect on GMCs in G2 phase. We speculated that this may be a preparation for the abnormal proliferation of GMCs, and the correctness of the analysis was verified by EdU experiments. Based on this, preventing excessive glucose from entering into cells is required for normal cell cycle and cell proliferation. BBR treatment not only increased the proportion of G1 phase cells but also reduced the proportion of S phase cells, and this tendency became more significant over time. This supports our hypothesis that major pathological responses are generated from HG‐induced abnormal GMC proliferation, and HG most likely stimulates GMCs to enter S phase, and increasing the total proportion of G1 phase cells by BBR treatment could exert a renoprotective effect. Therefore, it may be possible to interfere with the G1 and S phase distribution to ameliorate abnormal GMC proliferation by BBR treatment.

Studies have shown that GLUT1 is a vital regulator glucose homeostasis throughout the body, and its membrane recruitment is essential for the glucose uptake process.^34^, ^35^ In addition, a study indicated that the PI3K/Akt pathway functions as an upstream signalling pathway to stimulate the GLUT1 translocation to the cell surface in multiple tumour cells, thereby promoting glucose uptake.^31^, ^36^ During this process, GLUT1 translocation is mediated by AS160, which is activated through Akt phosphorylation.^29^ In the present study, BBR played a similar role to the PI3K inhibitor LY294002, which indicated that BBR can significantly inhibit HG‐induced PI3K‐p85 expression. Subsequently, the elevated expression levels of p‐Akt, p‐AS160 and membrane GLUT1 were significantly decreased after treatment with BBR, while their total protein expression changed undistinguishably. These results indicate that BBR reduces the membrane transport of GLUT1 protein when inhibiting PI3K/Akt/AS160 signal transduction rather than changing the level of total GLUT1 protein. This is consistent with the mechanism of action of GLUTs reported by authoritative research. Meanwhile, we found that mRNA had the same changing trend as proteins. These original observations provide the important knowledge that BBR inhibits the PI3K/Akt/AS160 signalling pathway to reduce the overexpression and membrane transport of GLUT1, as well as the abnormal cell cycle and GMC proliferation, eventually relieving the corresponding pathological changes.

In conclusion, the present study demonstrated that BBR could ameliorate DN progression to a certain degree. The mechanism may involve BBR inhibiting the activation of the PI3K/Akt/AS160/GLUT1 signalling pathway to prevent the HG‐induced abnormal GMC proliferation by retarding the cell cycle to remain at G1 phase. Therefore, regulating the cell cycle of GMCs is a potential therapeutic strategy, and BBR can also be considered as a promising therapeutic drug in the treatment of DN. Despite the advantages of BBR, its clinical application still has certain limitations, the most important of which is its low bioavailability due to its poor water solubility and gastrointestinal absorption. In view of this, in the future research, we will pay more attention to the structural modification and bioavailability of BBR while exploring its renoprotective mechanism.

## CONFLICT OF INTEREST

The authors declare no competing interests.

## AUTHOR CONTRIBUTIONS

**Wei‐Jian Ni:** Conceptualization (equal); funding acquisition (lead); project administration (equal); resources (supporting); validation (equal); writing – original draft (lead); writing – review and editing (equal). **Xi‐Mei Guan:** Conceptualization (equal); data curation (equal); formal analysis (equal); methodology (equal); software (equal); visualization (equal). **Jing Zeng:** Data curation (equal); investigation (equal); methodology (equal). **Hong Zhou:** Supervision (equal); validation (equal); visualization (equal). **Xiao‐Ming Meng:** Funding acquisition (equal); resources (equal); supervision (equal); writing – review and editing (equal). **Li‐Qin Tang:** Funding acquisition (equal); project administration (equal); resources (equal); writing – review and editing (equal).

## Supporting information

Table S1

## Data Availability

The data that support the findings of this study are available from the corresponding author upon reasonable request.
