# Supplementary material for: Berberine regulates mesangial cell proliferation and cell cycle to attenuate diabetic nephropathy through the PI3K/Akt/AS160/GLUT1 signalling pathway
Source: J Cell Mol Med. 2022 Jan 9;26(4):1144–55. doi: 10.1111/jcmm.17167 (PMC8831947; doi:10.1111/jcmm.17167)
Supplement: Supplementary file 1 — Table S1 [file JCMM-26-1144-s001.docx]

**Supplementary Table S1. Results of trypan blue staining of different times**

**Table S1A. Cell survival at 24 hours**

|  | **120μM** | **150μM** |  |
| --- | --- | --- | --- |
| **Ration of live cells (%)** | 78.0±3.0 | 67.0±1.5 |  |
| **Ration of dead cells (%)** | 22.0±3.0 | 31.0±5.0 |  |

**Table S1B. Cell survival at 36 hours**

|  | **90μM** | | **120μM** | **150μM** |
| --- | --- | --- | --- | --- |
| **Ration of live cells (%)** | | 75.0±4.0 | 67.0±1.5 | 60.0±3.0 |
| **Ration of dead cells (%)** | | 25.0±4.0 | 34.0±1.5 | 40.0±3.0 |

**Table S1C. Cell survival at 48 hours**

|  | **90μM** | **120μM** | **150μM** |
| --- | --- | --- | --- |
| **Ration of live cells (%)** | 36.0±5.5 | 14.0±0.5 | 7.0±1.0 |
| **Ration of dead cells (%)** | 65.0±5.5 | 87.0±0.5 | 93.0±1.0 |
